# An extra dimension in protein tagging by quantifying universal proteotypic peptides using targeted proteomics

**DOI:** 10.1038/srep27220

**Published:** 2016-06-06

**Authors:** Giel Vandemoortele, An Staes, Giulia Gonnelli, Noortje Samyn, Delphine De Sutter, Elien Vandermarliere, Evy Timmerman, Kris Gevaert, Lennart Martens, Sven Eyckerman

**Affiliations:** 1 VIB Medical Biotechnology Center, B-9000 Ghent, Belgium; 2 Department of Biochemistry, Ghent University, B-9000 Ghent, Belgium

## Abstract

The use of protein tagging to facilitate detailed characterization of target proteins has not only revolutionized cell biology, but also enabled biochemical analysis through efficient recovery of the protein complexes wherein the tagged proteins reside. The endogenous use of these tags for detailed protein characterization is widespread in lower organisms that allow for efficient homologous recombination. With the recent advances in genome engineering, tagging of endogenous proteins is now within reach for most experimental systems, including mammalian cell lines cultures. In this work, we describe the selection of peptides with ideal mass spectrometry characteristics for use in quantification of tagged proteins using targeted proteomics. We mined the proteome of the hyperthermophile *Pyrococcus furiosus* to obtain two peptides that are unique in the proteomes of all known model organisms (proteotypic) and allow sensitive quantification of target proteins in a complex background. By combining these ’Proteotypic peptides for Quantification by SRM’ (PQS peptides) with epitope tags, we demonstrate their use in co-immunoprecipitation experiments upon transfection of protein pairs, or after introduction of these tags in the endogenous proteins through genome engineering. Endogenous protein tagging for absolute quantification provides a powerful extra dimension to protein analysis, allowing the detailed characterization of endogenous proteins.

Epitope tags are widely used in cell biology and biochemical analysis^1^. They enable the detection of the protein in the cell and allow the purification of the associated protein complex. Many epitope tags exist, ranging from short peptide sequences (e.g., FLAG™^2^ and CaptureSelect™^3^) to intact proteins (e.g., Green Fluorescent Protein^4^, mutant BirA^5^ and Halo tag^6^). However, none of these tags allow for a direct quantification of the protein. Protein levels can be obtained by antibody-based approaches such as classical enzyme-linked immunosorbent assays (ELISA) experiments with theoretical detection and quantification limits in the zeptomolar concentration range^7^. In practice however, detection limits are often hampered by inadequate or non-specific antibodies, blocking of epitope sites by post-translational modifications (PTMs) or epitope masking by protein folding or interactors^8^. An alternative quantification strategy relies on the use of targeted mass spectrometry (MS) to monitor the expression level of a protein. Selective ion monitoring (SIM), selective reaction monitoring (SRM) and, more recently, parallel reaction monitoring (PRM) are targeted proteomics strategies that were developed to analyze specific proteins or protein modifications and are therefore increasingly used in translational research^9^. All of this resulted in the selection of SRM as the method of the year 2012 by the journal Nature Methods^10^. In these approaches, specific peptides are selected as representative reporter peptides for the protein of interest and allow either direct quantification, or indirectly by spiking a reference peptide that contains stable isotopic labels.

SRM experiments are typically performed on triple quadrupole instruments where the first (Q1) and the last (Q3) quadrupoles are used in a static manner to filter specific predefined precursor and fragment ions respectively, while in the second quadrupole (Q2) collision-induced dissociation of the precursor ion takes place^11^. In contrast to discovery MS, SRM does not rely on the acquisition of full-range m/z spectra but rather records the intensity over time for preselected m/z values. The combination of these predefined m/z values of the precursor and fragment ions are commonly denoted as transitions^11^.

In targeted proteomics, samples are first processed by the use of a robust protocol, followed by complete proteolytic digestion with trypsin or other proteases. Depending on sample complexity and target concentration, separation schemes can vary, with complex orthogonal fractionation strategies for sensitive detection of low abundant proteins^12^,^13^. Specific antibody-based peptide enrichment approaches such as Stable Isotope Standard Capture with Anti-Peptide Antibodies (SISCAPA)^14^, immunoaffinity depletion of abundant proteins or chromatographic optimizations are also used^15^,^16^.

Despite the clear use for targeted proteomics, especially as a verification step in biomarker studies to reduce the number of candidates coming from discovery screens^17^, assay development remains challenging, which recently culminated in an effort to standardize assay design and reporting^18^. As PTMs may interfere with the readout of specific SRM peptides, multiple peptides of a protein need to be monitored for a reliable protein assay. In addition, the selected peptides need to be proteotypic for the sample (i.e. unique for the protein given the background proteome in which the protein of interest is monitored), while sample complexity can impede signal-to-noise ratios (S/N) and liquid chromatography retention times^9^.

By using cleavable reporter peptides, equimolar amounts of a protein reference peptide and a universal peptide can be introduced in the sample, facilitating precise analysis^19^. Next to inaccurate stable isotope labeled reference peptide quantification^20^, incomplete digestion can also hamper robust quantification. In an attempt to correct for possible variation in digestion efficiency, labeled peptides are often synthesized with flanking sequences that mimic the actual protein sequence and thus approximate the endogenous tryptic context. This led to the emergence of advanced strategies over the last years, with QconCAT as the best-known example^21^,^22^. Peptides can be generated by classical synthesis or by expression of several concatenated peptides from a custom expression vector in cells cultured in media containing selected heavy labeled amino acids.

Co-immunoprecipitation (Co-IP) is a classical approach to show an interaction between two proteins. Transient expression of both tagged bait and prey proteins is typically used for these experiments, although the application of specific primary antibodies for both proteins is becoming increasingly popular and often required to ascertain an interaction on the endogenous level. In Co-IP, protein complexes are typically eluted from the purification beads and analyzed directly by Western blot which results in a semi-quantitative read-out.

In this report, we describe the selection of Proteotypic peptides for Quantification by SRM (PQS peptides) from the unique proteome of the hyperthermophile *Pyrococcus furiosus*. After several rounds of selection, two peptides were retained and evaluated for direct sensitive detection and quantification in complex lysates. These PQS peptides were also introduced as peptide tags in different target proteins, providing a universal quantitative read-out system for the tagged proteins. In addition, we performed binary protein-protein interaction (PPI) assays for different pairs of tagged proteins expressed from vectors in human cells, and for a binary interaction between two endogenous proteins after introduction of the PQS peptide sequences in the genome sequence of the target proteins.

## Results

### Initial selection of peptides based on *P. furiosus* proteome analysis

To enable sensitive SRM-based quantification using a protein tagging strategy, we first needed to define optimal peptides that could be developed into a universal SRM assay. We rationalized that such peptides should adhere to the following criteria. 1) The peptides need to show good LC-MS(/MS) properties, including ionization and fragmentation, to maximize assay sensitivity. Ideally, such peptides can be detected in complex samples such as digested whole cell lysates without the need for fractionation prior to detection. 2) The SRM peptides are unique or proteotypic for a wide range of commonly used model organisms in life sciences research. 3) The peptides allow for quantification in complex samples. 4) The peptides should be generated very efficiently by trypsin, minimizing potential quantification deviations when performing Stable Isotope Dilution (SID) experiments. 5) Finally, heavy labeled counterparts should be straightforward to produce and easy to handle (e.g. not prone to precipitation).

**Figure 1:**
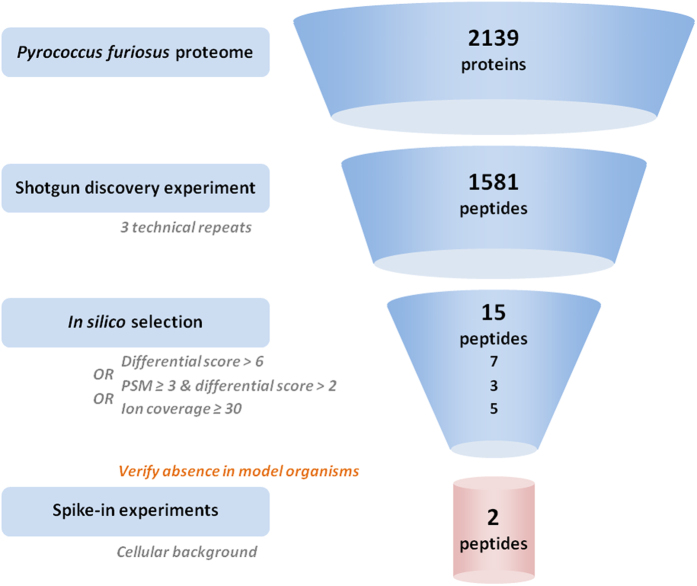
Selection of universal proteotypic peptides for quantification by SRM. The *P. furiosus* UniProt database contained 2,139 proteins. By shotgun discovery proteome analysis on the Agilent complex proteomics standard, 1,581 unique candidate peptides were retrieved. Three *in silico* selection approaches reduced this number to 15. After experimental validation of the *in silico* predicted shortlist, two peptides were retained. PSM: peptide-spectrum match.

To maximize the chance of retrieving unique peptides with all of these properties, we reasoned that a proteomic analysis of *P. furiosus*, a well-characterized hyperthermophile archaeon which thrives at an optimum growth temperature of 100 °C^23^, should lead to rapid identification of peptides with the desired specifications. The remarkable characteristics of *P. furiosus* are encoded in a genome that is drastically different from most other species which leads to an overlap of merely seven tryptic peptides upon comparison with human protein tryptic peptides^24^. Despite this discrepancy, Vaudel *et al.* showed that peptides from both proteomes show a very similar behavior in LC-MS systems^24^. Therefore, the Agilent complex proteomics standard (i.e. a lyophilized total soluble *P. furiosus* extract) was digested, fractionated and analyzed by LC-MS/MS. This standard is predicted to contain the majority of the approximately 2,000 known proteins that *P. furiosus* encodes for and led here to the identification of 1,581 unique peptide sequences, originating from 582 unique proteins, that were retained for further selection and validation. An overview of the complete peptide triage workflow is provided in Fig. 1.

To narrow down the number of suitable peptides, the data obtained from the *P. furiosus* discovery experiments were further processed to create a shortlist of peptides that show optimal LC-MS characteristics. To this end, average matched intensities were calculated for each peptide that uniquely matched a single protein. Differential scores were subsequently calculated for all peptides based on the Huber median and mean absolute deviation (MAD) values as defined in experimental procedures. These differential scores were then averaged for each peptide across the three discovery experiments. A second calculation was done for the fragmentation efficiency, represented by the fragment ion coverage. For each identified peptide, the number of annotated ions for every spectrum were summed and averaged over the three discovery experiments.

**Figure 2:**
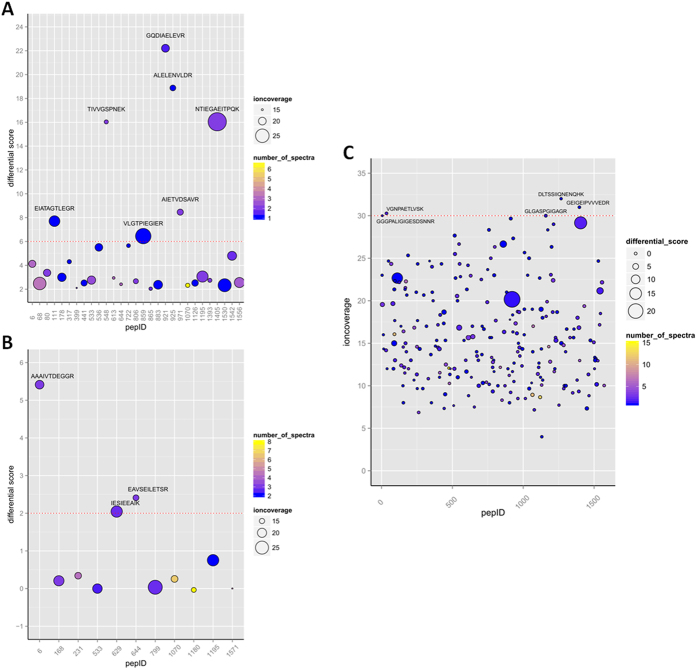
PQS Peptide selection strategy. Panel (**A**) shows the selection of a first set of peptides with consistent identification in the three replicates and with a differential score exceeding six (red dotted line). After filtering for suitable SRM peptides, this resulted in a set of seven peptides (labeled data points). Panel (**B**) shows a second selection of peptides where only peptides identified with at least three spectra were considered for the differential score calculation. After filtering for SRM application, only peptides with a differential score >two (red dotted line) and identified in all three replicates were retained, which in turn gave a set of three new peptides (labeled). The last approach panel (**C**) considered the average ion coverage as a selection criterion together with the consistent identification in all three replicates. Only peptides that show an ion coverage of 30 or higher (red dotted line) were selected. From this analysis, five peptides (labeled) were added to the total list.

Following calculations, the peptides were filtered in an elimination process where peptides with at least one of the following criteria were discarded: 1) peptides that contain methionines or cysteines (oxidation effects), 2) peptides that show missed cleavages, 3) peptides that start with glutamine (non-quantitative pyro-glutamic acid formation), 4) peptides that show consecutive prolines (elution peak broadening) and 5) peptides that are shorter than 9 amino acids. From the remaining list, a first set of peptides was selected by considering only peptides having a differential score >6 in all three replicates which resulted in a set of 7 peptides (indicated by selection method 1 in Table 1, Fig. 2A). A second selection of peptides was obtained by only considering peptides identified with at least 3 spectra for the differential score calculation. After elimination, only peptides with a differential score >2 and identified in the three replicates were retained, which in turn gave a set of 3 new peptides (indicated by selection method 2 in Table 1, Fig. 2B). The last approach considered the average ion coverage as a selection criterion combined with identification in all three replicates. Only peptides showing an ion coverage of 30 or higher were selected. This resulted in a selection of 5 peptides (indicated by selection method 3 in Table 1, Fig. 2C). Taken together, these 3 approaches led to a list of 15 potential peptides (Table 1). Next, we verified the uniqueness of the peptides irrespective of any cleavage rules by mapping the peptides back to protein databases. This analysis was done for all *Eukaryota*, and for all strains of *E. coli.* None of these peptide sequences were found in these protein databases, which include the vast majority of model organisms.

### SRM-based evaluation of selected peptides

First, the selected peptides were optimized for LC-SRM by deriving the top 8 transitions with their best collision energy condition. The list of *in silico* predicted peptides was evaluated by spike in of a dilution series of this 15-peptide mix (Table 1) in a tryptic digest of a human colorectal carcinoma cell line lysate (HCT116, ATCC) followed by LC-SRM using optimized settings. Peptides that were readily detected in this complex matrix were further considered as the best candidates for use as universal PQS peptides. From this analysis, the two peptides that showed the highest intensity and the most equal distributed transitions were retained (Supplementary Figure S-1). These PQS peptides were further designated PQS1 (EAV) and PQS2 (GLG).

### Sensitivity determination of PQS1 and PQS2 in a cellular background

**Figure 3:**
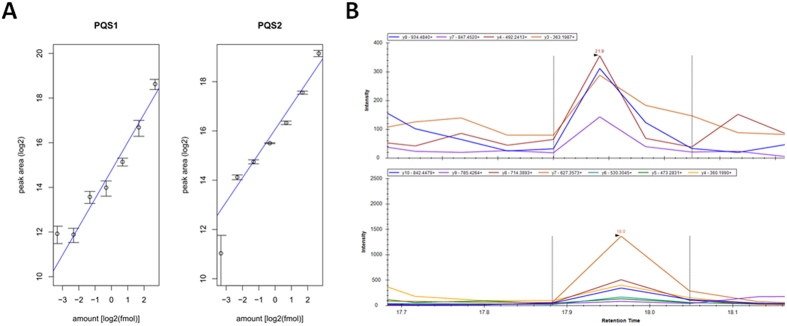
Limit Of Detection (LOD) values for PQS1 (EAVSEILETSR) and PQS2 (GLGASPGIGAGR). A dilution series of the peptide was spiked in a tryptic digest of HCT116 cells and transitions were monitored. (**A**) Calibration curve for each peptide depicting the average for 3 replicate runs with error bars of ±1 SD. (**B**) LOD determination for PQS1 (top) and PQS2 (bottom) by the S/N method. For PQS1, the calibration curve (**A**) bends at 200 amol on column, but the signal still has a S/N > 3 at 100 amol (**B**). The peak area of PQS2 for the analysis of 100 amol is out of the calibration curve (**A**) and the transitions are not distinguishable anymore. The signal at 200 amol is still on the curve and has a S/N > 10 (**B**).

To investigate the sensitivity associated with the highest ranked peptides EAVSEILETSR (PQS1) and GLGASPGIGAGR (PQS2), we spiked a dilution series of these peptides in a tryptic digest of HCT116 cells. Here, peptide levels varied over a dilution range from 6.4 fmol to 0.1 fmol detectable peptide in 0.5 μg of HCT116 proteome digest (on column; quantities reported throughout this report refer to detectable peptide injected on the column, Fig. 3). Triplicate runs were averaged and plotted to determine the Limit of Detection (LOD) in a complex proteome background. For PQS1, a LOD of 100 amol was determined when an S/N higher than 3 was considered. Compensation for the non-linearity of the calibration curve resulted in a LOD of 200 amol. For PQS2, both approaches resulted in the same LOD of 200 amol. These values are in accordance to other SRM initiatives^16^,^25^.

### Evaluation of PQS peptides upon transient expression of tagged proteins

**Figure 4:**
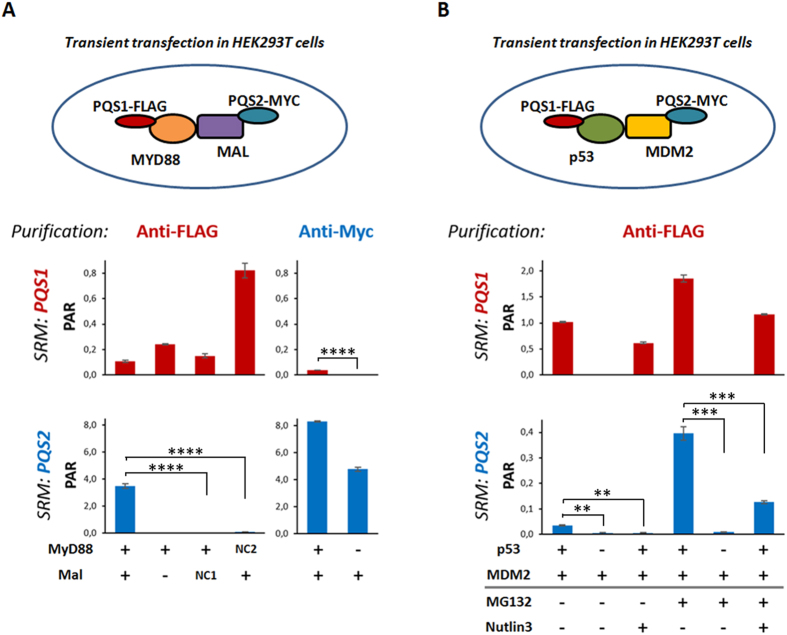
Use of PQS peptides to study binary interactions between tagged proteins upon expression in human cells. Peak Area Ratios to heavy (PAR) of summed transitions for PQS1 and PQS2 are shown for three experimental setups in HEK293T cells. In each sample, stable isotope labeled peptide were added to a final injected amount of 80 fmol. Each depicted sample was run in technical triplicate, error bars represent mean error values. A representative experiment is shown for 3 independent biological repeats. (**A**) Interaction between Mal and MyD88 as shown by PQS peptide quantification. Different combinations of FLAG-PQS1-MyD88, Myc-PQS2-Mal and mock interactors (Myc-PQS2-MARK3 (NC1) and FLAG-PQS1-Ras (NC2)) were transfected. After immuno-precipitation using anti-FLAG or anti-Myc followed by on-bead tryptic digest, the levels of PQS1 and PQS2 were quantified by SRM. Independent t-test; all p values <0.0001. (**B**) PQS peptide quantification for the interaction between p53 and MDM2. Different combinations of FLAG-PQS1-p53 and Myc-PQS2-MDM2 were expressed in human cells. The interaction was monitored in absence or presence of the proteasome inhibitor (MG132) or a small-molecule inhibitor of the p53 - MDM2 interaction (Nutlin3). A significance drop in signal by Nutlin3 addition could be observed both in absence and presence of MG132 (Independent t-test, p values: 0.0012 and 0.0006 in absence and presence of MG132, respectively).

To further demonstrate the utility of these universal peptides for SRM analysis, we generated expression vectors that contain an N-terminal module which consists of the FLAG tag combined with the PQS1 peptide, and vectors with the N-terminal Myc-PQS2 peptide tag. Both PQS peptides were embedded in an optimal tryptic context as predicted by the cleavage prediction with decision trees (CP-DT) algorithm (LTLIFR-PQS1-FAYLYD and VAEAYR-PQS2-FLETEN)^26^. The well-studied interaction pair MyD88 and Mal from the TLR4 signaling pathway^27^ was then cloned to obtain N-terminally tagged constructs with FLAG-PQS1 and Myc-PQS2 respectively. Upon transfection in HEK293T cells, the complex was purified using either anti-FLAG or anti-Myc antibody coupled to magnetic beads prior to on-bead digestion. Peak areas for each peptide behave as expected, corroborating the use of the PQS peptides as SRM readout (Fig. 4A). Interestingly Myc-PQS2-Mal readouts showed significantly higher peak area ratios when normalized to their heavy labeled counterparts, which indicates that more Mal proteins were enriched when compared to MyD88 proteins. This is in accordance with literature stating Mal homodimerization can lead to the formation of potential binding platforms on the top and the side of the Mal dimer that bind MyD88^28^. All combinations showed a robust transition pattern (highest CV value detected: 8.73%). To verify there was no non-specific binding of the peptides to the antibodies or beads we included a mock-interaction pair for each peptide. No signal could be observed when an unrelated prey (MARK3; NC1) or bait (HRAS; NC2) was used (Fig. 4A).

In addition to Mal-MyD88, we next tested the PQS peptides as readout for the LCP2-GRAP2 interaction involved in T-cell signaling^29^. Figure S-2 shows the reciprocal detection of the interaction using both FLAG-based immunoprecipitation (IP) and Myc-based IP of the associated baits. We also explored the clinically relevant binary interaction between p53 and its critical modulator MDM2^30^. We monitored MDM2 retrieval by following the PQS2 peptide under basal conditions and under different treatment conditions that affect either the stability of the proteins (MG132) or the interaction between bait and prey (Nutlin3^31^). Nutlin3 is a well-characterized small molecule that disrupts the autoregulator feedback loop between p53 and MDM2 by occupation of the p53 binding site of MDM2^31^,^32^,^33^. Indeed, we were able to show significant alterations in the monitored MDM2 levels in presence of Nutlin3 (independent t-test, p values: 0.0012 and 0.0006 in absence and presence of MG132 respectively, Fig. 4B).

### Integration of PQS peptides in endogenous proteins by genome engineering

**Figure 5:**
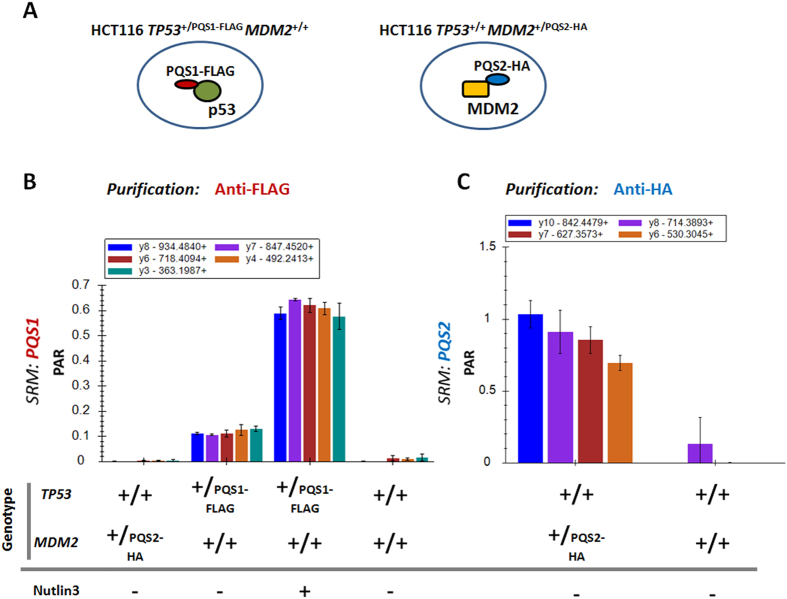
Affinity purification followed by SRM analysis of PQS peptides introduced in endogenous proteins in human HCT116 cells. (**A**) Schematic representation of the two engineered HCT116 cell lines employed in this experiment. One of two alleles encodes for the tagged version of the protein. The PQS1-3xFLAG combinatorial tag was introduced on the C-terminus of the endogenous p53 protein resulting in HCT116 TP53^+/PQS1-FLAG^ MDM2^+/+^ cells, while the PQS2-3xHAtag was introduced on endogenous MDM2 resulting in HCT116 TP53^+/+^ MDM2^+/PQS2-HA^ cells. (**B**) Peak area ratio (PAR) measurement of 5 transitions of the PQS1 peptide after enrichment with anti-FLAG antibody. (**C**) PAR of 4 transitions of the PQS2 peptide after anti-HA mediated enrichment. The plots depict the peak area ratio of the light peptide to the heavy counterpart (PAR; final amount: 15 fmol injected).

We further explored the use of the PQS peptides by their introduction at the C-terminus of endogenous proteins. We used recombinant adeno-associated virus (rAAV) in combination with Clustered Regularly-Interspaced Short Palindromic Repeats (CRISPR)/Cas9 to generate a cell line that expresses an endogenous p53 protein containing a C-terminal combinatorial 3xFLAG-PQS1 tag (HCT116 *TP53*^+/PQS1-FLAG^). The tryptic context was also introduced to ensure optimal release upon tryptic digestion. We also generated a cell line containing the 3xHA-PQS2 tag fused to the C-terminus of MDM2 (HCT116 *MDM2*^+/PQS2-HA^) (Fig. 5A). Cell lines were validated by PCR analysis, DNA sequencing, Southern blot and Western blot (Supplementary Figures S-3 and S-4 respectively). Upon specific pull down of the bait protein (anti-FLAG for p53 and anti-HA for MDM2) a clear signal was observed in the enriched samples from engineered cell lines (p53 by PQS1, MDM2 by PQS2), while no signal was observed in control samples (Fig. 5B,C). Next, we assessed dynamic interaction profiles by increasing p53 protein levels through addition of Nutlin3. As expected, Nutlin3 treatment leads to increased levels of p53 and thus the PQS1 peptide (Fig. 5B).

**Figure 6:**
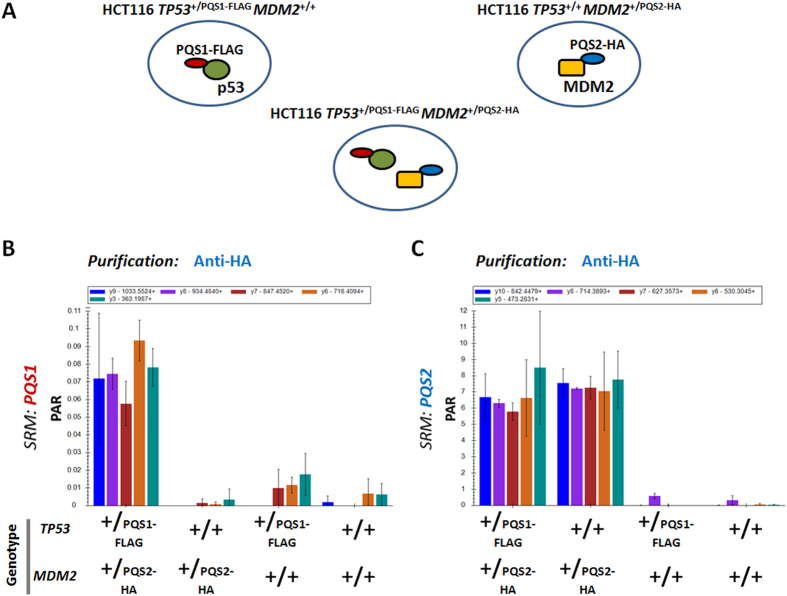
Detection of the p53-MDM2 interaction in HCT116 cells expressing tagged endogenous proteins. (**A**) Schematic representations of the engineered cell lines used for this experiment. The same cell lines were employed as in Fig. 5, with the addition of cell line expressing both MDM2 and p53 with a specific tag on the endogenous protein. (**B,C**) show peak area ratios (PAR) for the PQS1 and PQS2 peptide respectively after enrichment with anti-HA. The plots indicate the peak area ratio of the light peptide to the heavy counterpart (PAR, 5 fmol heavy peptide injected). 5 transitions were followed for every peptide in each run.

In a next step we monitored the interaction between p53 and MDM2. To this end, we created an additional cell line where the 3xFLAG-PQS1 tag sequence was introduced on endogenous p53 in the background of the engineered MDM2 cell line, resulting in HCT116 *TP53*^+/PQS1-FLAG^ *MDM2*^+/PQS2-HA^ cells (Fig. 6A). This engineered cell line allows quantitative endogenous Co-IP analysis. Because of a high turnover rate, wild-type p53 and MDM2 are typically expressed at low endogenous levels in most cultured cell lines including HCT116 cells. By using the PQS peptides as a proxy, we were able to detect the interaction in presence of MG132 when enriching for the complex by the HA-tag fused to MDM2 linked by the PQS2 peptide in the engineered HCT116 cells (Fig. 6B,C).

**Figure 7:**
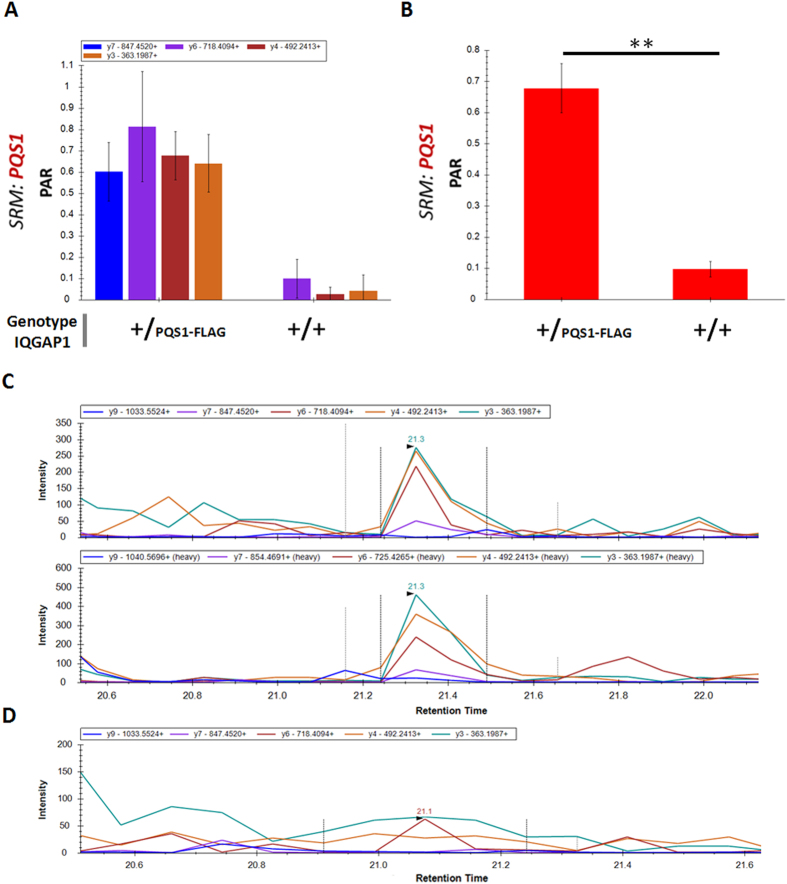
Detection and quantification of PQS1 for endogenous IQGAP1 in engineered HCT116 *IQGAP1*^+/PQS1-FLAG^ cells. (**A**) Peak area ratio (PAR) for individual transitions of the PQS1 peptide compared to wild-type HCT116 cells. Heavy peptide was added to the sample to a final amount of 15 fmol heavy peptide on column. (**B**) PAR for the PQS1 peptide in HCT116 *IQGAP1*^+/PQS1-FLAG^ cells and background signal in a wild-type HCT116 population. Each sample depicts three technical repeats with a final amount of 15 fmol heavy PQS1 peptide. Independent t-test, p value: 0.0021. (**C**) Typical chromatograms of monitored transitions for the light (top) and heavy (bottom) PQS1 peptide. (**D**) Typical chromatogram of parental (wild type) HCT116 cells. Three transitions were omitted due to background interference as described in the experimental procedure section.

To explore direct detection and quantification of endogenous expressed proteins in a complex sample without the necessity to enrich or use tedious fractionation strategies, we used another engineered cell line wherein we introduced the 3xFLAG-PQS1 combinatorial tag C-terminally in the IQGAP1 protein (HCT116 *IQGAP1*^+/PQS1-FLAG^; Fig. 7A). IQGAP1 is a key mediator of cytoskeletal rearrangements and is thought to play an important role in several cancer types by stimulating cell mobility and invasion^34^. Because of the involvement of IQGAP1 in cancer development, SRM experiments were described before to monitor expression levels in complex backgrounds with detection of 8.6 fmol using a SISCAPA approach^35^. For our assay, we developed a straightforward protocol which consists of minimal sample handling steps (see experimental procedures section). By spiking in heavy labeled peptide we were able to directly detect and quantify the PQS1 SRM peptide (Fig. 7B–D). Using the built-in peak detection feature in Skyline combined with manual curation of the signal, an average peak area ratio to heavy (15 fmol) of 0.679 (CV value: 11.45%) was obtained, which translates to 10.18 fmol light peptide measured in 5.14 μg of sample. By including 7 transitions for detection and using eventually 4 transitions for analysis of both light and heavy peptide we are able to identify the correct peaks with high confidence (typical chromatograms are depicted in Fig. 7C,D).

## Discussion

In this work we present small peptides flanked by optimal tryptic contexts that are characterized by optimal MS properties and thus allow SRM-based detection and quantification of proteins upon incorporation of the peptide sequence as a tag to the protein. The selected peptides are universally applicable being proteotypic for all *Eukaryota* and all strains of *E. coli*, thus covering a wide scope of model organisms used in life sciences. We were able to readily apply the defined peptides in overexpression experiments where Co-IP of selected protein pairs was studied. Moreover, we demonstrate the direct quantitative analysis of a human protein upon introduction of a PQS peptide by genome engineering using only one SRM assay in contrast to a classical setup in which a labor intensive SRM assay is developed for each protein, frequently with a low success rate. In addition, we show the use of these peptides for endogenous Co-IP experiments upon introduction in p53 and its interaction partner MDM2, which shows a high sensitivity of the PQS peptides. Variation in tryptic digest was minimized by providing these peptides in an optimal trypsin context that was derived by the aid of the CP-DT algorithm, alleviating the need for (complex) normalization approaches incorporated in the heavy labeled counterparts^19^,^22^.

Clearly, a possible disadvantage of our approach is the need to fuse the PQS peptide(s) to the protein(s) of interest. This can cause artifacts as the PQS tag may perturb protein folding, or have unexpected effects on transcription or translation. We focused all genome engineering efforts on the C-terminus of the native protein, as we rationalized the risk of interference with regulatory elements is lower at this end of the protein^36^,^37^. Nevertheless, at this point it is hard to predict interference effects of fused sequences on protein expression, folding or interactions and use of tagging by genome editing should be approached empirically. The incorporation of PQS peptides in an ‘Inntag’^38^ design process may minimize these artifacts.

Classical Co-IP approaches typically rely on semi-quantitative Western analysis as a read-out^39^. The LUMIER approach where a luciferase-tagged prey is captured by an epitope-tagged bait is an alternative binary approach in which the luminescence read-out is accurately quantified^40^. However, bait quantification is completely lacking in this system. The PQS peptides presented here provide a simple and efficient way to quantify bait and prey both in lysates and enriched fractions. This information can be used to help elucidate the stoichiometry of the complex, and can provide information on the affinity of the associations^41^. Moreover, the actual protocol used to obtain the protein complexes can be optimized using the PQS peptides by monitoring losses in steps of the pull-down process. The work from Hakhverdyan *et al.* clearly demonstrates a need for lysis optimization or even complementary lysis conditions to obtain a comprehensive view on a protein complex^42^. Use of the PQS peptides can now be extended to binary reference sets as described for the binary PPI approaches^43^. This will allow a side-by-side comparison between classical Co-IP pull down experiments and binary approaches such as yeast two-hybrid and MAPPIT^44^,^45^. While most of the published Co-IP data involves forced expression of bait and prey proteins, the revolution of genome engineering tools now allows the rapid engineering of epitope tags in endogenous proteins as demonstrated in this work^37^. Further optimization and streamlining of the SRM protocol for detection of the PQS peptides may allow the assessment of small molecule effects on protein expression or on PPIs as shown for the nutlin3 effect on the p53 - MDM2 interaction. Although true high-throughput screens may be too ambitious for this technology because of the sequential nature of the analysis, focused screens on lead(-optimized) compounds are clearly within reach.

While we rely on the combinatorial use of well-defined affinity sequences to enrich for the protein(s) of interest which causes our tag sequences to grow considerable in length, it is theoretically possible to only depend on the PQS peptide for both enrichment and detection by generating (polyclonal) antibodies against the sequence^46^,^47^. This would enable inline enrichment of the peptides, boosting the sensitivity even further as described earlier in the SISCAPA approach^14^,^48^. To push accurate quantification even further, existing experimental setups such as QconCAT or universal cleavable reporter peptides can be incorporated when synthesizing heavy labeled versions of the peptides to provide more accurate quantification of peptides. Additionally, sensitivity can be further enhanced by tagging proteins with concatenated versions of the peptide. While the focus of this work lies on proteomics and protein interactions we envision an extra applicability of PQS peptides for bioproduction purposes where these peptides could provide an elegant read-out system to monitor and optimize the production of biologicals. In addition to the sensitive PQS peptides we were able to identify, this work also provides a robust workflow to identify sensitive and proteotypic peptides from any given host organism. Theoretically, this workflow could be expanded to search optimal PQS peptides in a wide collection of organisms, which may lead to the identification of more and perhaps better PQS peptides useful for detection and quantification of proteins expressed at very low levels in complex samples.

### Experimental procedures

#### *In silico* selection of candidate SRM peptides

For initial selection of the PQS peptides, we first analyzed the Agilent complex proteomics standard as a source for *P. furiosus* proteins using a classical shotgun approach. The exact experimental details can be found in the supplementary information.

Identification data from the *P. furiosus* proteome was retrieved from ms_lims^49^ and used to compute metrics for the selection of optimal SRM peptides. For every identified peptide, the average matched intensity was computed in each of the three discovery experiments as follows. First, absolute intensities of all matched singly and doubly charged b and y fragment ions were summed for each peptide-spectrum match (PSM). Subsequently, these matched intensity values were averaged across the number of PSMs for each identified peptide sequence to yield an average matched peptide intensity. Only peptides that matched uniquely to a single protein entry were considered. Moreover, peptides that matched to a protein that was identified with at least three peptides were retained for downstream evaluation. For each protein, the location and scale of the average matched peptide intensity distributions were estimated through the median and mean absolute deviation (MAD), respectively. These values were calculated from the list of average matched peptide intensities for each protein by robust Huber statistics as implemented in the ‘*huber*’ function of the R ‘*MASS*’ package. This function applies iterative winsorization to reduce the effect of outliers on the distribution of values^50^. The *k* parameter that determines the outlier range was set to 1.2. The Huber median and MAD values were then used to compute a differential score for each peptide using the formula 
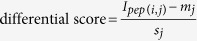
; where *I*_*pep(i, j)*_ is the average matched peptide intensity for peptide *i* of protein *j*, *m*_*j*_ is the huber median of the average matched peptide intensities for protein *j*, and *s*_*j*_ is the huber MAD of the average matched peptide intensities for protein *j*. An additional calculation was done for the fragmentation efficiency, indicated by the fragment ion coverage. For each identified peptide, the number of annotated ions for every spectrum were summed and averaged over the three discovery experiments.

Uniqueness of the final selection of *P. furiosus* peptides was then verified by mapping the peptide sequences back, irrespective of any cleavage rules, to the corresponding UniProt protein databases of all Eukaryota (UniProt release-2015_09; 181,063 protein sequences), and all *Escherichia coli* strains (UniProt, release 2015_09; 23,008 protein sequences) with DBToolkit^51^.

#### Evaluation of predicted PQS peptides

The 15 best *in silico* predicted PQS peptides were synthesized in-house using standard solid-phase Fmoc chemistry on a Syrol peptide synthesizer (Biotage) and purified by RP-HPLC. For the initial evaluation of *in silico* predicted peptides, 100 fmol of each peptide was analyzed on the TSQ Vantage LC-SRM system operated as described in the supplementary experimental procedures section. Here, the collision energy was experimentally optimized through Skyline. From this analysis, the 8 most efficient transitions of these 15 peptides were derived. To challenge the peptides in a complex sample, a dilution series (to final injection amounts of 25, 50, 100, 150, 250 and 400 fmol) of a mix containing the 15 peptides (Table 1) was spiked into a tryptic digest of HCT116 cells (ATCC) (1 μg on column) and analyzed with timed SRM. For the final ranking of the best peptides, two criteria were taken into account: 1) intensity of the total peak area of all transitions and 2) an optimal equal distribution of the peak areas for each transition.

All Skyline files used for obtaining data presented in this work, both targeted and *P. furiosus* discovery experiments were uploaded using Panorama^52^ and are publicly available at https://panoramaweb.org/labkey/PQSpeptides.

#### Limit of detection experiment

The two most intense PQS peptides, designated PQS1 and PQS2, were spiked in a tryptic digest of HCT116 cells (0.5 μg injected) in separate dilution series (final injected amounts: 0.1, 0.2, 0.4, 0.8, 1.6, 3.2, and 6.4 fmol) for each peptide. To each sample, the appropriate heavy labeled counterpart was added to a final injection amount of 5 fmol as reference for quantification, instrument stability and peak identification facilitation. The final LOD values correspond to the lowest detectable amount of light peptide (i.e. S/N > 3) in the sample series for each peptide. Standard deviations were calculated based on the extracted mean standard error values from Skyline.

#### *In silico* prediction of the optimal tryptic context

The two selected PQS peptides were *in silico* evaluated for their tryptic propensity when inserted into the human proteome. We therefore first determined all tryptic peptides from the human proteome with the aid of DBToolkit^51^. Next, each human tryptic peptide was added both before and after the PQS peptide of interest. The resulting merged peptide library was subsequently submitted to the CP-DT algorithm^26^. CP-DT is based on a decision tree ensemble learned on publicly available peptide identifications and calculates the probability of cleavage by trypsin of the tryptic position within each merged peptide. For each PQS peptide, the merged peptides were then ranked according to the probability of cleavage by trypsin. The top ranked merged peptides, the peptides with the highest probability of cleavage by trypsin, were used for further analysis.

#### cDNA plasmid generation

cDNA coding for proteins of interest was obtained from the human ORFeome collection (v8.1, http://horfdb.dfci.harvard.edu/)^53^. Constructs were inserted into pMet7-FLAG-PQS1 or pMet7-Myc-PQS2 backbone plasmids by Gateway cloning, fusing the affinity sequence and corresponding PQS peptides flanked by their optimal tryptic context to the N-terminus of the protein-coding cDNA. All final constructs were verified by restriction digest and Sanger sequencing. Detailed outlines regarding transfection, cell lysis and affinity purification can be found in the supplementary experimental procedures.

#### Endogenous protein tagging based on recombinant adeno-associated virus and CRISPR/Cas9

Recombinant adeno-associated virus (rAAV)-based genome editing was performed on HCT116 cells cultured in McCoy’s 5A medium in presence of 10% fetal bovine serum (Gibco). Virus production was performed in AAV-293 cells (Agilent) by Polyethylenimine (PEI) co-transfection of a modified pAAV-MCS plasmid (Agilent), hereafter referred to as targeting vector and the pDG vector (Plasmidfactory). For generation of HCT116 *TP53*^+/PQS1-FLAG^ and HCT116 *IQGAP1*^+/PQS1-FLAG^ engineered cell lines, the final targeting vector consisted of two homology regions of each approximately 1,000 bp length spanning the C-terminus of the target gene embedding the PQS1 peptide flanked by an optimal tryptic context and a 3xFLAG-tag. After the stop codon, a floxed neomycin selection cassette (Genscript, custom DNA synthesis) was inserted for selection purposes. Homology regions were picked up by genomic PCR on HCT116 DNA. Separate inserts were simultaneously ligated using In-Fusion cloning kits (Clontech). All primer sequences and DNA constructed by DNA synthesis can be found in Supplementary Table S-1. For generation of HCT116 MDM2^+/PQS2-HA^ cells, the PQS2 peptide was inserted and the 3xFLAG sequence was exchanged for a 3xHA-tag. 24 h after transfection, the medium was refreshed and after 48 h of additional incubation viral stocks were prepared using the AAV purification ViraKit (Virapur) according to the protocol provided by the manufacturer. Engineering of p53 was performed by a combination of rAAV and CRISPR/Cas9 whereby rAAV infection was done one day after Cas9 transfection using FugeneHD (Promega). CRISPR guide RNAs (gRNAs) (Supplementary Table S-2) were used based on *in silico* activity prediction using the online Zhang Lab CRISPR design tool (http://crispr.mit.edu/) and constructed in *Streptococcus pyogenes* Cas9 in the pSpCas9(BB)-2A-Puro vector (PX459, Addgene) according to the protocol published by Ran *et al.*^54^. Activity of the gRNAs was assessed experimentally by transfecting the different constructs and cleavage of the target region was assessed using the Surveyor^®^ mutation detection kit (Integrated DNA Technologies) (Supplementary Figure S-5). Only the most potent gRNA was further used for the generation of knock-in cell lines. The HCT116 *TP53*^+/PQS1-FLAG^ MDM2^+/PQS2-HA^ cells were generated by additional genome engineering using the rAAV-CRISPR/Cas9 combination for TP53 on HCT116 MDM2^+/PQS2-HA^ cells. The detailed genome engineering workflow and clonal screening can be found in the supplementary experimental procedures.

#### Sample preparation from engineered cell lines

For direct detection of the SRM peptides in cell lysates without prior enrichment, subconfluent 145 cm^2^ petri dish engineered clonal HCT116 populations were lysed in 100 μl 20 mM ammonium acetate by freeze-thawing after detaching the cells with cell dissociation buffer (Gibco). Digestion occurred overnight by addition of trypsin equal to 1% of the total protein concentration of the sample as determined by Bradford assay. Prior to transferring the sample to a MS vial, Trifluoroacetic Acid (TFA) was added to a final concentration of 0.1% and the sample was centrifuged at 16,000 g for 10 min to remove any debris.

Protocols regarding endogenous affinity purification experiments are described in supplementary experimental procedures.

## Additional Information

**How to cite this article**: Vandemoortele, G. *et al.* An extra dimension in protein tagging by quantifying universal proteotypic peptides using targeted proteomics. *Sci. Rep.* **6**, 27220; doi: 10.1038/srep27220 (2016).

## Supplementary Material

Supplementary Information

## Figures and Tables

**Table 1:** Overview of the 15 *in silico* predicted peptides and their corresponding ranking for intensity and equal distribution of transitions when spiked in a tryptic HCT116 digest.

Rank in digest	Peptide	Abbreviation	Selection set
1	EAVSEILETSR	EAV	2
2	GLGASPGIGAGR	GLG	3
3	TIVVGSPNEK	TIV	1
4	NTIEGAEITPQK	NTI	1
5	VLGTPIEGIER	VLG	1
6	ALELENVLDR	ALE	1
7	AIETVDSAVR	AIE	1
8	IESIEEAIK	IES	2
9	GEIGEIPVVVEDR	GEI	3
10	GGGPALIGIGESDSNNR	GGG	3
11	AAAIVTDEGGR	AAA	2
12	VGNPAETLVSK	VGN	3
13	DLTSSIIQNENQHK	DLT	3
14	EIATAGTLEGR	EIA	1
15	GQDIAELEVR	GQD	1

The last column depicts by which procedure the peptides were selected. The indicated abbreviation is the code the peptides were given during the experimental evaluation of the peptides.
