# Supplementary material for: An extra dimension in protein tagging by quantifying universal proteotypic peptides using targeted proteomics
Source: Sci Rep. 2016 Jun 6;6:27220. doi: 10.1038/srep27220 (PMC4893672; doi:10.1038/srep27220)
Supplement: Supplementary Information [file srep27220-s1.pdf]

Supporting information

An extra dimension in protein tagging by quantifying universal proteotypic peptides using targeted proteomics.

Giel Vandemoortele[1,2§], An Staes[1,2§], Giulia Gonnelli[1,2§], Noortje Samyn[1,2], Delphine De Sutter[1,2], Elien Vandermarliere[1,2], Evy Timmerman[1,2], Kris Gevaert[1,2], Lennart Martens[1,2] and Sven Eyckerman[1,2]*

[1]VIB Medical Biotechnology Center, B-9000 Ghent, Belgium

[2]Department of Biochemistry, Ghent University, B-9000 Ghent, Belgium

[§]These authors contributed equally to this work

Running title: Optimal peptides for Selected Reaction Monitoring (SRM) of tagged proteins

*Correspondence to Prof. Dr. Sven Eyckerman

VIB Medical Biotechnology Center

A. Baertsoenkaai 3, B-9000 Ghent, Belgium

Tel: +32-9-264.92.18

Fax: +32-9-264.94.90

Email: sven.eyckerman@vib-ugent.be

Supplementary experimental procedures

LC-MS/MS analysis of the P. furiosus proteome

To obtain suitable PQS peptides we used the Agilent complex proteomics standard as a source for *P. furiosus* proteins. After suspension of 500 µg standard in 50 µl water, 50 mM NH_4HCO_3 pH 8.0, 4 mM DTT, and 50% trifluoroethanol (TFE) was added to 200 µg of the *P. furiosus* lysate followed by 1 h incubation at 60°C. After reduction and denaturation, iodoacetamide was added to a final concentration of 15 mM and the lysate was subsequently alkylated for 1 h in the dark at 30°C prior to lowering the concentration of TFE in the sample to 5% using 50 mM NH_4HCO_3 pH 8.0. 10 µg trypsin (Promega) was added and digestion occurred overnight at 37°C. The digestion was inhibited by the addition of formic acid to a final concentration of 5% and the peptide mixture was then vacuum-dried in a vacuum concentrator. Hereafter, the dried sample was re-dissolved in 500 µl solvent A (10 mM ammonium acetate pH 5.5 in water/acetonitrile (ACN) 98/2 (v/v)) and methionines were oxidized by the addition of H_2O_2 to a final concentration of 0.5% in the HPLC's autosampler followed by 30 min incubation at 30°C prior to injection on the RP-HPLC column. Peptides were separated on a 2.1 mm I.D. x 150 mm column (Zorbax®, 300 SB-C18 Narrowbore, Agilent Technologies, Waldbronn, Germany) through an 1100 Agilent series HPLC. A 140 min gradient was used in which the peptides were loaded for 10 min in solvent A. Peptide separation was performed by a 1% increase of solvent B (water/ACN, 30/70 (v/v), 10 mM ammonium acetate pH 5.5) per min until 100% solvent B was reached, followed by a re-equilibration for 20 min with solvent A. Peptide fractions separated by 20 min were pooled, vacuum-dried and re-dissolved in 20 µl loading solvent (0.1% trifluoroacetic acid (TFA), 2% ACN).

The pooled fractions were injected into the LC-MS/MS system consisting of an Ultimate 3000 RSLC nanoLC instrument (Dionex) in-line connected to an LTQ Orbitrap Velos (Thermo Fisher Scientific). The sample mixture was loaded on a trapping column (made in-house, 100 µm I.D. x 20 mm, 5 µm beads C18 Reprosil-HD, Dr. Maisch). After flushing from the trapping column, the sample was loaded on a reverse-phase column (made in-house, 75 µm I.D. x 150 mm, 3 µm beads C18 Reprosil-HD, Dr. Maisch).

Peptides were loaded using solvent A' (0.1% formic acid, 2% ACN) and separated with a linear gradient from 2% solvent B' (0.1% formic acid and 80% ACN) to 55% solvent B' at a flow rate of 300 nl/min followed by a wash reaching 100% solvent B'.

The mass spectrometer was operated in data-dependent acquisition mode, automatically switching between MS and MS/MS acquisition for the ten most abundant peaks in a given MS spectrum. In the LTQ-Orbitrap Velos, full scan MS spectra were acquired in the Orbitrap at a target value of 1E6 with a resolution of 60,000. The ten most intense ions were then isolated in the linear ion trap with a target value of 5E4 and a dynamic exclusion of 20 s and were fragmented in the HCD cell. From the MS/MS data in each LC run Mascot Generic Files were created using Distiller software (version 2.4.3.3, Matrix Science, www.matrixscience.com/Distiller). These peak lists were then searched with the Mascot search engine (Matrix Science) using the Mascot Daemon interface (version 2.4, Matrix Science)[1]. The search was performed against the *P. furiosus* database (from UniProt, release-2012_06, containing 2139 proteins). Cysteine carbamidomethylation and methionine oxidation were set as fixed modifications and pyroglutamate formation of amino terminal glutamine as variable modification. Mass tolerance on peptide ions was set to 10 ppm (with Mascot's C13 option set to 1), and the mass tolerance on peptide fragment ions was set to 20 millimass units (mmu). The peptide charge was set to 1+, 2+, 3+ and the instrument setting was put on ESI-QUAD. The enzyme was set to trypsin allowing for one missed cleavage, and cleavage was allowed when arginine or lysine was followed by proline. Only peptides that were ranked first and scored above the threshold score, set at 99% confidence, were withheld (average FDR across the three discovery experiments: 2.30%). All data was processed and managed by the ms_lims database[2].

TSQ Vantage LC-SRM instrument settings and SRM data analysis

For SRM analyses a TSQ Vantage instrument (Thermo Fisher Scientific) was used coupled online to an Ultimate 3000 RSLC system (Thermo Fisher Scientific), using the nano-ESI source. The voltage was set to 1,300 V and the capillary temperature to 275°C. 5 µl sample was injected and loaded at 10 µl/min in loading solvent (2/98 ACN/H_2O, 0.1% TFA). Binding on the trapping column (made in-house, 100 µm

I.D. x 20 mm, 5 µm beads C18 Reprosil-HD, Dr. Maisch) and flushing away the salts occurred for 4 min using loading solvent, after which the trapping column was set in line with the analytical column (made in-house, 75 µm I.D. x 150 mm, 3 µm beads C18 Reprosil-HD, Dr. Maisch). Peptides were eluted at 300 nl/min using an ACN gradient of 30 min to 35% solvent B'. Next, flushing with 90% solvent B' occurred for 1 min, followed by an equilibration with solvent A' for 9.5 min. For all runs, a cycle time of 5 s was used with a Q1 window of 0.2 Th.

All SRM data analysis and quantitation was performed using Skyline[3]. For all analyses, 7 and 8 transitions were monitored for the PQS1 and PQS2 peptide respectively. For the analysis of samples for engineered cell lines, a transition was left out if the signal when it had a larger variability compared to the other transitions. At least 4 transitions were retained to perform further data analysis. All figures containing experimental data that rely on transient expression or data based on the use of engineered cell lines are representative for three biological repeats each performed in technical triplicate. The full list of transitions monitored for PQS1 and PQS2 can be found in Supplementary Table S-3.

Transient overexpression experiments

One day prior to transfection 1.2 x 10^6 HEK293T cells cultured in standard conditions using DMEM (Gibco) and 10% Foetal Calf Serum (FCS, Gibco) were seeded in 6 well plates for each transfection condition (Nunc). Constructs were transfected using polyethylenimine (PEI). 24 h post-transfection, cells were detached from the culture well plates and lysed using 50 mM Tris-HCl pH 8, 200 mM NaCl, 2 mM EDTA, 1% NP-40 (IGEPAL, Sigma), 0.5% deoxycholate, 0.05% sodium dodecyl sulfate (SDS), complete proteasome inhibitor tablet (Roche), 1 mM NaF, 1 mM Na_3VO_4 and 20 mM β-glycerophosphate. Each lysate was centrifuged at 16,000 g for 15 min at 4°C and the supernatant was transferred to protein LoBind tubes (Eppendorf). Samples were incubated for 2 h at 4°C with 20 µl MyOne™ Streptavidin T1 Dynabeads® (Invitrogen) prebound with 2 µl monoclonal anti-FLAG® BioM2 antibody (Sigma) or 20 µl Protein G Dynabeads® prebound with 2 µg anti-Myc tag antibody (in-house prepared) was added for FLAG-tag or Myc-tag pull-out respectively. After 2 h of incubation, beads were magnetized and washed twice with lysis buffer. Beads were suspended in 10 mM Tris-HCl pH 8 before

0.25 µg trypsin (Promega) was added and digestion occurred overnight at 37°C. Magnetization of the sample after trypsin digestion allowed for removal of the beads.

Epitope tagging workflow and clonal screening

Three days post-infection with the rAAV packaged targeting constructs, HCT116 cells were selected for homologous recombination events by culturing in presence of G418 (Invitrogen) during 2 weeks. Following G418 selection, single cells were seeded in 96-well plates. After reaching 70% confluency, half of the cultures were lysed and gDNA was prepared using phase lock gel Eppendorfs (5Prime) according to the manufacturer protocol. PCR based screening consisted of two junction PCRs. In both PCRs, one of the primers mapped on the homology region incorporated in the rAAV-packed repair template, whereas the second primer mapped on the flanking genomic region not embedded in the rAAV repair template (Supplementary Table S-1). gDNA that was positive for these PCRs was retained. To assess single integration of the tag sequence, Southern blotting of the neomycin resistance cassette was performed after the gDNA was digested with EcoRI (New England Biolabs) by generating an α-^{32}P CTP (Perkin Elmer) labeled probe using Rediprime II DNA labeling system (GE Healtcare) (Supplementary Table S-1 and Supplementary Figure S-3). Neomycin selection cassettes in retained clonal populations were removed by transfection of TAT-Cre recombinase (Excellgen) after which single cells of the transfected population were seeded in 96 well plates. When clonal populations reached the subconfluent stage, half of the cells in each clonal population were used for a novel round of screening. In this stage, Southern blot was used to determine excision of the neomycin selection cassette (Supplementary Figure S-3). Finally, insertion of the desired sequence was verified by Sanger sequencing of the engineered C-terminus of the tagged gene (primers in Supplementary Table S-1) and Western blotting (Supplementary Figure S-4). Retained clonal populations showing aberrant doubling time or morphology when compared to wild type HCT116 cells were discarded nonetheless.

Endogenous affinity purification on engineered HCT116 cells.

Engineered HCT116 clonal populations were cultured in 145 cm^2 surface area petri dishes (Nunc) until

cells reached a subconfluent state. Cells were lysed using 50 mM Tris-HCl pH 8, 200 mM NaCl, 1% NP-40 (IGEPAL, Sigma), 0.5% deoxycholate, 0.05% SDS, Complete proteasome inhibitor tablet (Roche), 1 mM NaF, 1 mM Na_3VO_4 and 20 mM β-glycerophosphate. After centrifugation of the lysate at 16,000 g at 4°C for 15 min, a solution of 20 µl MyOne™ Streptavidin T1 Dynabeads® (Invitrogen) prebound with 2 µl Monoclonal ANTI-FLAG® BioM2 antibody (Sigma) or 20 µl Protein G Dynabeads® prebound with 2 µg anti-HA high affinity antibody (Sigma) was added for FLAG-tag or HA-tag pull-out respectively. After 2 h of incubation, beads were magnetized and washed twice with lysis buffer. On-bead trypsin digestion was performed in 10 mM Tris-HCl pH 8. In case of Nutlin3 treatment of the cells, 20 µM of the compound (Selleck Biochem) was added for 24 h prior to cell lysis. For the endogenous p53-MDM2 interaction, 10 µM MG132 (Calbiochem) was added to the cell for 6 h prior to harvest and cell lysis.

Supplementary tables and figures

Table S-1. Primers used for genome engineering experiments. HR: homology region; SB: Southern blot. Fragments were generated by DNA synthesis where indicated.

PCR	Forward primer	Reverse primer
TP53 5'HR	GCGGCCGCAAACGCGTGTTCCGAGAGCTGAATGAGG	CTTTTGAATTCGTTGGGCCCGCTGTCTGAGTCAGGCCCTTC
TP53 3'HR	CTCGAGTTATCGATTGACATTCTCCACTTCTTGTTC	GCTTTTGCTAGCTTGGTACCAAATGCAGATGTGCTTGCAG
TP53 junction 5'HR	ATCAGCCAAGATTGCACCAT	GGGGAACTTCCTGACTAGGG
TP53 junction 3'HR	GGGAGGATTGGGAAGACAAT	CCAGTCTCCAGCCTTTGTTC
TP53 surveyor	ACATATTTGCATGGGGTGTG	CCTAGAATGTGGCTGATTGTAAAC
TP53 insert seq.	TCCACAGGAAGCCGAGCTGTC	GGGGCTCGACGCTAGGATCTG
MDM2 5'HR	Custom gBlock DNA synthesis (Integrated DNA technologies) (1)	
MDM2 3'HR	TAATCGATAACTCGAGTAGTTGACCTGTCTATAAGAG	GGCCGCTTTTGCTAGCGCACTCTAGCATGGGTGATG
MDM2 junction 5'HR	TTGAGCCTTTAAGGGAGTGG	GGGGAACTTCCTGACTAGGG
MDM2 junction 3'HR	GGGAGGATTGGGAAGACAAT	CAAAAATCTGAGGCCAGGTG
MDM2 insert seq.	GCCCTGCCCAGTATGTAGAC	GGAGTTGGTGTAAAGGATGAGC
IQGAP1 5'HR	Custom gBlock DNA synthesis (Integrated DNA technologies) (2)	
IQGAP1 3'HR	Custom gBlock DNA synthesis (Integrated DNA technologies) (3)	
IQGAP1 junction 5'HR	GTGTAGTGGCGTGTTCTCCA	GGGGAACTTCCTGACTAGGG
IQGAP1 junction 3'HR	GGGAGGATTGGGAAGACAAT	AGGCTCAGCAGCATGATTTC
IQGAP1 insert seq.	TGCCTAGCGTGGAACTTTTC	GCTGTGAGGTGCTTCTTTCC
Neomycin SB probe	TGCTCCTGCCGAGAAAGTAT	GCGATGCAATTTCCTCATTT

(1) MDM2 5’HR

CGCAAACGCGTAAAGGGCCCGTGTGTATGGTTAAAATACCTAAAATCTATTCTTTTAGTAAATTTCCAGTATACCAATATATAA
CTAACTATAGTCCTCATGCTGTTTACAGTGACTATTTTATATAAAACATGAAACACTGAATATTGAGCCCTATGATATACTTTAC
CTTAGACATAGCAAAGTTGCTAGCATTCCTGTGACTGAGCAGTTAAAGGGTTACAGAAACTGACTGTGTGTCTTATTTCATTG
AAGGACTATTGGAAATGCACTTCATGCAATGAAATGAATCCCCCCCTTCCATCACATTGCAACAGATGTTGGGCCCTTCGTGA
GAATTGGCTTCCTGAAGATAAAGGGAAAGATAAAGGGGAAATCTCTGAGAAAGCCAAACTGGAAAACTCAACACAAGCTGA
AGAGGGCTTTGATGTTCCTGATTGTAAAAAAACTATAGTGAATGATTCCAGAGAGTCATGTGTTGAGGAAAATGATGATAAA
ATTACACAAGCTTCACAATCACAAGAAAGTGAAGACTATTCTCAGCCATCAACTTCTAGTAGCATTATTTATAGCAGCCAAGAA
GATGTGAAAGAGTTTGAAAGGGAAGAAACCCAAGACAAAGAAGAGAGTGTGGAATCTAGTTTGCCCCTTAATGCCATTGAA
CCTTGTGTGATTTGTCAAGGTCGACCTAAAAATGGTTGCATTGTCCATGGCAAAACAGGACATCTTATGGCCTGCTTTACATGT

GCAAAGAAGCTAAAGAAAAGGAATAAGCCCTGCCCAGTATGTAGACAACCAATTCAAATGATTGTGCTAACTTATTTCCCCGA
ATTCGGGAAGTTCC

(2) IQGAP1 5’HR

CGCAAACGCGTAAAGGGCCCAGACCACTCAGATTTCTTAAACTTGTTCAGGACAATGGTGAAAGTAGCAGGAAAAGCGGGA
GCATTGCAGTCAGATCTGGATTTATCGCCTCCATCTCTGTCCTGTTGTGCAAGTCATTTAACATCTCTGAGTCTCAGTAGCTCAT
CTTTAAAGTTAGAATAATCGCACCTCATAAAAATTTTTGTGAGGACTTTAAAAGATACTATGTAATGTGCTTACACAATGCCAA
GCACATGGCAGACACCTAATAAAACATCAAGTTCTCTCTTTTCCCCTTGCCTTCGGGATAGAAAGTGTTCTCATATGGAATAGA
AATTAAGAATCTATCAGATAGTATTAAGGTGACATTACTAGCCTTATCTCATTTCCCCGTGCTTAGTATATTTCATCATAGGTTT
AAATGCTTCACCTTTTCCAGATTTATTAAAACTAGTGACCCATAACATGGATGAAATGGGAAAATGTCATTGTGACTTCTGAAA
TAAAAAGCTTAGATGTGTGTGGCAATTGAACATGTTTCTTTTTCTTTCCATGTATGATGAATTTTGATATGAACAACATGACTTC
CTAACTCCAAATCAACCAGAAGCCTCCCTTGGCCAATGTGCCCTATTGCTCCAGCTCTCAGGCTGGTTGTGCAGTGGGTAGTT
GCCTAGCGTGGAACTTTTCTTTCGCCCCAGTTCACATCTGGCACACAAGGCAGGCCTCAGTCCATCTGGATCCCTCCCTACATT
GTGTCCTTTTTCCTTGTTCCACAGACTTGATTAACTGTCAAAATGTTTTGTTTTGTTTTGTTTTGTTTTGTTAATAGGACCTGCTG
CAGCTACAGTATGAAGGAGTTGCAGTCATGAAATTATTTGATAGAGCTAAAGTAAATGTCAACCTCCTGATCTTCCTTCTCAAC
AAAAAGTTCTACGGGAAGGAATTCGGGAAGTTCC

(3) IQGAP1 3’HR

TAATCGATAACTCGAGTAATTGATCGTTTGCTGCCAGCCCAGAAGGATGAAGGAAAGAAGCACCTCACAGCTCCTTTCTAGGT
CCTTCTTTCCTCATTGGAAGCAAAGACCTAGCCAACAACAGCACCTCAATCTGATACACTCCCGATGCCACATTTTTAACTCCTC
TCGCTCTGATGGGACATTTGTTACCCTTTTTTCATAGTGAAATTGTGTTTCAGGCTTAGTCTGACCTTTCTGGTTTCTTCATTTTC
TTCCATTACTTAGGAAAGAGTGGAAACTCCACTAAAATTTCTCTGTGTTGTTACAGTCTTAGAGGTTGCAGTACTATATTGTAA
GCTTTGGTGTTTGTTTAATTAGCAATAGGGATGGTAGGATTCAAATGTGTGTCATTTAGAAGTGGAAGCTATTAGCACCAATG
ACATAAATACATACAAGACACACAACTAAAATGTCATGTTATTAACAGTTATTAGGTTGTCATTTAAAAATAAAGTTCCTTTAT
ATTTCTGTCCCATCAGGAAAACTGAAGGATATGGGGAATCATTGGTTATCTTCCATTGTGTTTTTCTTTATGGACAGGAGCTAA
TGGAAGTGACAGTCATGTTCAAAGGAAGCATTTCTAGAAAAAAGGAGATAATGTTTTTAAATTTCATTATCAAACTTGGGCAA
TTCTGTTTGTGTAACTCCCCGACTAGTGGATGGGAGAGTCCCATTGCTAAAATTCAGCTACTCAGATAAATTCAGAATGGGTC
AAGGCACCTGCCTGTTTTTGTTGGTGCACAGAGATTGACTTGATTCAGAGAGACAATTCACTCCATCCCTATGGCAGAGGAAT
GGGTTAGCCCTAATGTAGAATGTCATTGTTTTTAAAACTGTTTTATATCTTAAGAGTGCCGCTAGCAAAAGCGGCC

Table S-2. gRNA sequences tested for p53 C-terminal CRISPR/Cas9 constructs. Left: final guide sequences. Right: synthesized oligonucleotide sequences used for annealing and subsequent cloning in the pSPCas9(BB)-2A-Puro vector (PX459, Addgene). The guide sequence used in engineering efforts of the tagged p53 and double tagged p53-MDM2 cell line is underlined.

**#**	**gRNA sequence**	**oligonucleotide sequences**
<u>1</u>	<u>ACGCACACCUAUUGCAAGCA</u>	CACCGACGCACACCTATTGCAAGCA<br>AAACTGCTTGCAATAGGTGTGCGTC
2	GGAGAAUGTCAGUCUGAGUC	CACCGGGAGAATGTCAGTCTGAGTC<br>AAACGACTCAGACTGACATTCTCCC
3	AAACUCAUGUUCAAGACAGA	CACCGAAACTCATGTTCAAGACAGA<br>AAACTCTGTCTTGAACATGAGTTTC
4	GGGGGUGGGAGGCUGUCAGU	CACCGGGGGGTGGGAGGCTGTCAGT<br>AAACACTGACAGCCTCCCACCCCCC
5	UCUCCCUCCCCUGCCAUUUU	CACCGTCTCCCTCCCCTGCCATTTT<br>AAACAAAATGGCAGGGGAGGGAGAC

Table S-3. Monitored transition list for PSQ1 and PQS2. Depending on sample complexity, not all transitions were taken into account for data analysis. A minimum of 4 transitions were retained for all experiments.

**EAVSEILETSR**		**GLGASPGIGAGR**	
Y9	1033.5524+	Y10	842.4479+
Y8	934.4840+	Y9	785.4264+
Y7	847.4520+	Y8	714.3893+
Y6	718.4094+	Y7	627.3573+
Y5	605.3253+	Y6	530.3045+
Y4	492.2413+	Y5	473.2831+
Y3	363.1987+	Y6	360.1990+
		Y3	303.1775+

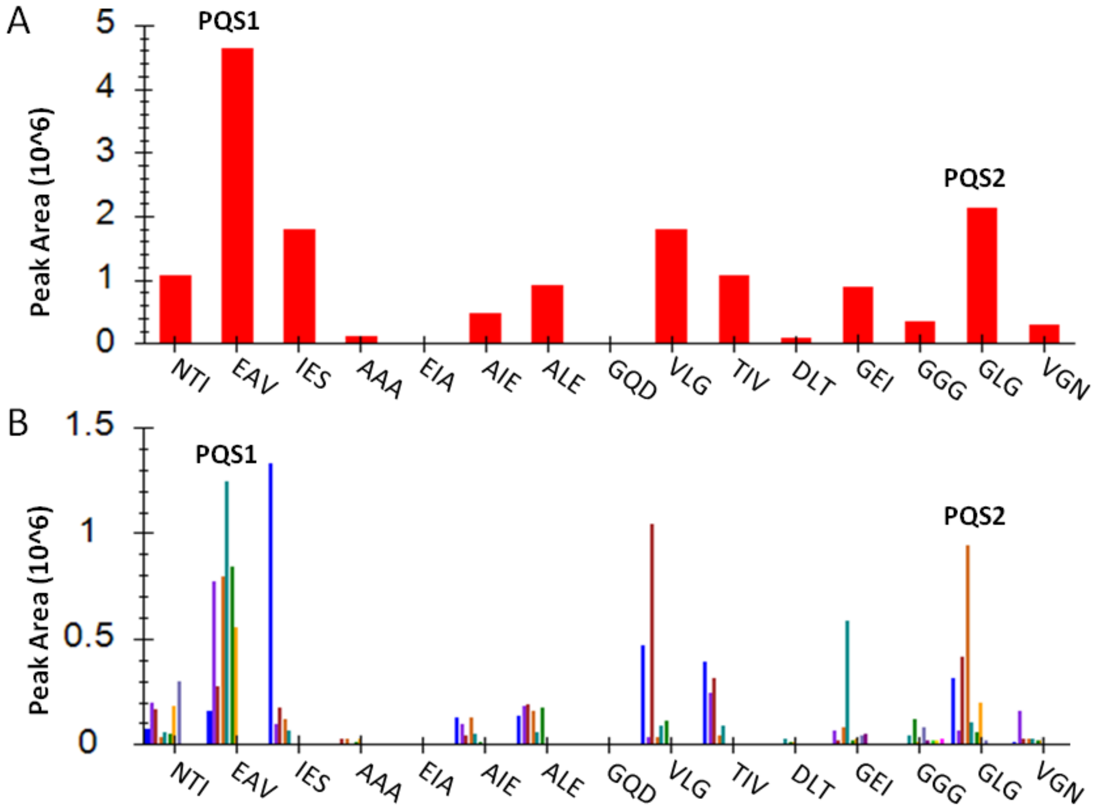

Figure S-1: Selection of the best peptides out of the in silico selected peptides. All panels show the peak area of either the total of all transitions (A) or each transition separately for each of the *in silico* predicted peptides (B). The graphs depicts each peptide when spiked-in to a final on column amount of 250 fmol. Based on both total peak area of the peptides and equal peak area distribution, the two best peptides were selected: EAVSEILETSR (PQS1), GLGASPGIGAGR (PQS2).

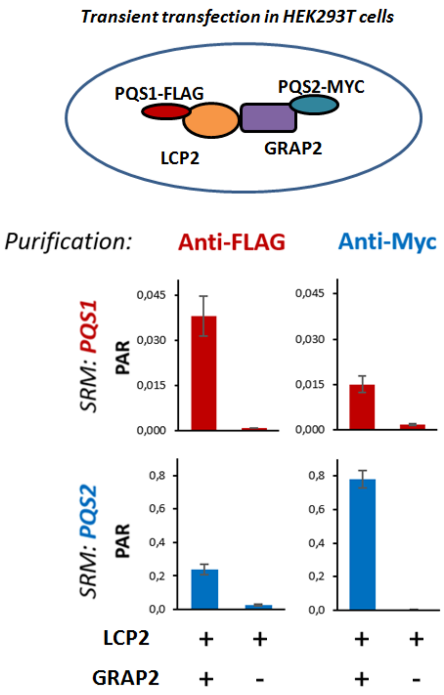

Figure S-2. SRM analysis of PQS peptides for the interaction between LCP2 and GRAP2. Different transfection combinations of FLAG-PQS1-LCP2 and Myc-PQS2-GRAP2 were performed. PAR: Peak area ratio to heavy. Graph depicts three technical replicates.

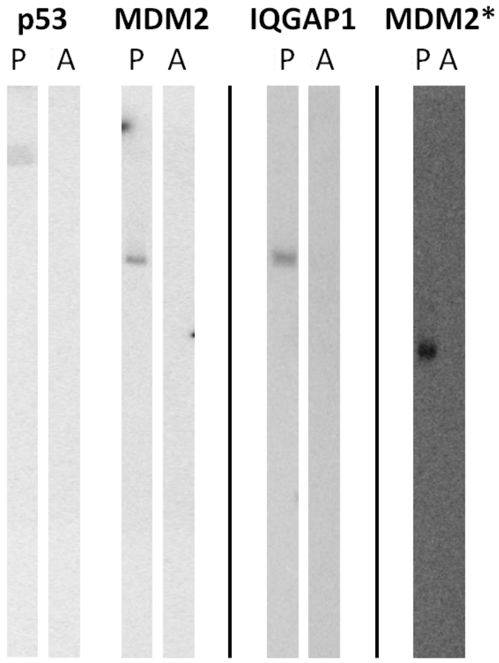

Figure S-3. Southern blot results for rAAV engineered HCT116 cells to assess single integration of the tag sequence. Neomycin resistance cassette α-^{32}P CTP probe was added to gDNA of modified clonal populations that was digested with EcoRI. White spacing is used to indicate non-neighboring lanes from the same blot. Black separation lines are indicative for different blots. The MDM2* indicated lanes show the Southern blot after MDM2 tagging in the double tagged cell line originating from the depicted p53 cell line. P and A: Southern blotting results prior to and after Cre-lox mediated neomycin selection cassette removal respectively.

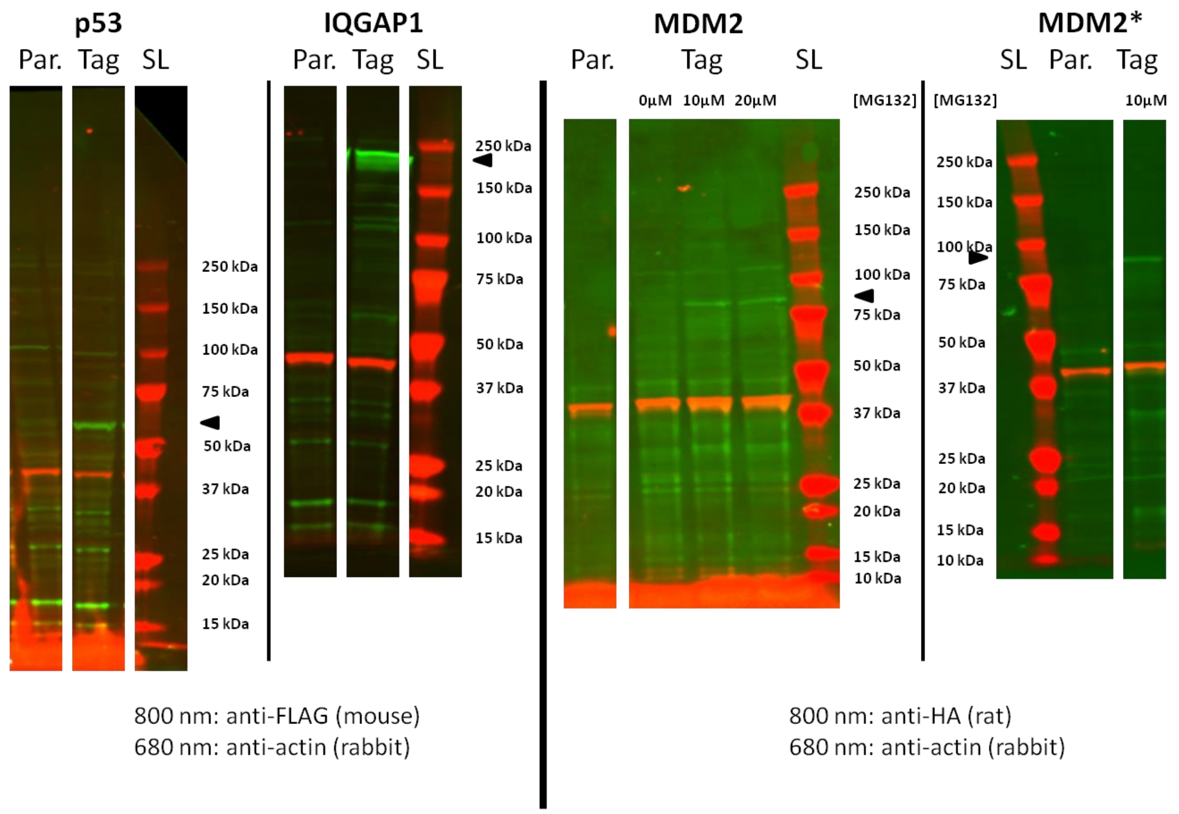

Figure S-4: Western blot results for rAAV engineered HCT116 cells. White spacing is used to indicate non-neighboring lanes from the same blot. Black separation lines are indicative for different blots. Protein headers of each panel indicate which protein was tagged and analyzed on the corresponding western blot. Black triangles indicate the position of the tagged protein on the corresponding blot. The MDM2* indicated lanes show the western blot after MDM2 tagging in the double tagged cell line originating from the depicted p53 cell line in the outer left panel. Tagged MDM2 could not be detected in lysates unless MG132 was added, as indicated by the concentrations above the appropriate lanes. In each lane, lysate corresponding to a total protein amount of 50 µg was loaded on the SDS-PAGE gel. Par.: parental HCT116 cells; Tag: clonal population of tagged HCT116 cells; SL: smartladder (Eurogentec).

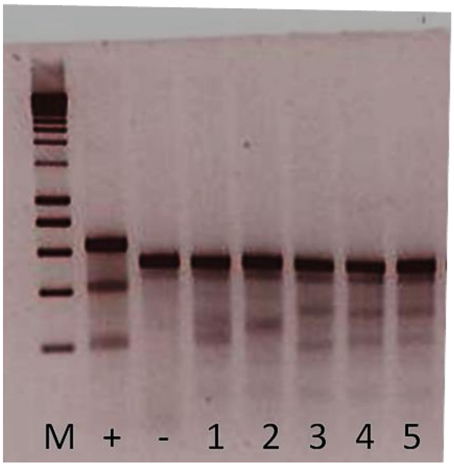

Figure S-5. Surveyor based read-out of p53 C-terminally directed gRNA potency in HEK293T cells. +: positive control Surveyor kit (G/C control); -: negative control (gDNA non-transfected cells subjected to Surveyor nuclease); 1-5: different gRNA constructs as annotated in supplementary table S-2. Similar potency could be observed for all tested gRNA sequences.

Supplementary references

1. Perkins, D. N., Pappin, D. J., Creasy, D. M. & Cottrell, J. S. Probability-based protein identification by searching sequence databases using mass spectrometry data. *Electrophoresis* **20**, 3551-3567 (1999).
2. Helsens, K. *et al.* ms_lims, a simple yet powerful open source laboratory information management system for MS-driven proteomics. *Proteomics* **10**, 1261-1264 (2010).
3. MacLean, B. *et al.* Skyline: an open source document editor for creating and analyzing targeted proteomics experiments. *Bioinformatics* **26**, 966-968 (2010).
